# Family climate influences next-generation family business leader effectiveness and work engagement

**DOI:** 10.3389/fpsyg.2023.1110282

**Published:** 2023-06-15

**Authors:** Stephen P. Miller

**Affiliations:** Family Enterprise Center, Kenan-Flagler Business School, University of North Carolina at Chapel Hill, Chapel Hill, NC, United States

**Keywords:** emotional intelligence, family business, family climate, leadership effectiveness, next-generation leader, responsibility, open communication, work engagement

## Abstract

Effective next-generation leadership is central to the multi-generational survival of family businesses. This study of 100 next-generation family business leaders found that business-owning families that openly express their opinions, take time to listen to each other, and squarely address difficult issues positively influence the development of the emotional and social intelligence competencies in next-generation family leaders that drive their leadership effectiveness. That kind of open and transparent communication in the family also makes it more likely next-generation leaders will be held accountable for their leadership performance by others, which increases the degree to which they are positively engaged with their work in the family firm. On the other hand, the results suggest that senior-generation family leaders who lead autocratically, a leadership style often observed in entrepreneurs who found family firms, make it less likely that next-generation family leaders will learn the emotional and social intelligence competencies that predict their leadership effectiveness. The study also found that autocratic senior-generation leaders negatively affect next-generation leader self-efficacy and make it less likely that others will hold them accountable, which limits their engagement with work in the family business. One of the study’s most important findings is that next-generation leader acceptance of personal responsibility for their leadership behaviors and results serves as a mediator through which the nature of the family climate influences their leadership effectiveness and work engagement. This suggests that while the nature of family relationships may make it easier or more difficult, next-generation family leaders have ultimate control over the development of their leadership talent and the inspiration, enthusiasm, energy, and pride they feel when working in the family business.

## Introduction

Next-generation leaders of family firms face challenges not experienced by leaders of other types of businesses. In addition to managing the business, they must also negotiate the dynamics of the overlapping family and ownership systems that characterize family enterprises (Gersick et al., 1997). They must do this with the knowledge that the multi-generational survival rate for family firms is low. It is estimated that only 30% of family enterprises pass from the first to the second generation, 12% from the second to the third, and 4% from the third to the fourth (Poza and Daugherty, 2014).

The low survival rate of family firms is an important issue for family owners and for the economies of the communities and countries where they are located. A recent study found that 54% of GDP is generated by the 32.4 million family businesses located in the United States and that those firms employe 59% of the private workforce (Pieper et al., 2021). In most other countries, family businesses are even more important, generating over 75% of GDP. It is estimated that over 80% of the companies in the world’s free economies are owned or controlled by families (Poza and Daugherty, 2014), including 20% of the Fortune Global 500 (The Economist, 2014).

Effective next-generation leadership is critical for those business-owning families who want the businesses they have worked hard to create to prosper through multiple generations of family ownership. In one study, family business owners identified weak next generation leadership as one of the three most significant threats to the long-term success of their firms (Ward, 1997). Noted family enterprise expert James Hughes stresses the importance of developing human and intellectual capital to the preservation and growth of family wealth (Hughes, 2004). Sadly, when most business-owning families plan for succession in the family firm they prioritize estate tax planning, ownership transfer, and investment policy, and fail to create plans for developing the human capital needed to lead the business into the future (Morris et al., 1997).

So what can business-owing families do to foster the development of the leadership talent next-generation family members need to effectively lead the family business? How can next generation family leaders learn leadership skills, fully engage with their work in the family firm, and earn the respect of those whom they will lead by overcoming the challenge of “living in the shadow” of a mother, father, grandparent, or other family member who founded or grew the family enterprise? Some research has been done on leadership development in large public companies (Mccall et al., 1988; Conger and Benjamin, 1999), but there is very little research on how leadership talent is developed in family-owned enterprises. Much of the literature on family business emphasizes the importance of effective next-generation leadership, but more rigorous research is needed on factors that influence the development of the leadership skills they need to effectively lead their family firms (Fiegener et al., 1994; Cabrera-Suárez, 2005). This paper reports the results of a quantitative study of 100 next-generation family business leaders and 350 members of their firms and families who observe their leadership behaviors designed to address this gap in the literature.

The paper begins with a review of the primary theories that informed the development of hypotheses, followed by a detailed description of research methods and data analysis. The paper continues with a discussion that includes interpretation of results, limitations of the study, and avenues for further research; and concludes with contributions to the literature and implications for family business practice.

### Family business theories

Systems theory, the resource-based view, the stewardship perspective, and agency theory are the most frequently used theoretical frameworks used to understand the unique attributes of family businesses. Family business systems theory is based on the dynamics created by the interaction among the family, ownership, and business subsystems, which overlap and are interdependent (Gersick et al., 1997). The resource-based view theorizes that unique and often idiosyncratic characteristics of family firms such as patient capital, transfer of knowledge from one generation to the next, rapid decision making, and concentrated ownership are employed to create competitive advantage (Habbershon and Williams, 1999). The stewardship perspective is based on the idea that family business owners are often committed to a mission that extends beyond the goal of making money and self-interest and feel a responsibility to leave a positive legacy future generations can build on. Agency theory holds that the costs of operating a family business can be either higher or lower than those in a non-family business because ownership and management positions are often held by the same person or group of family members (Poza and Daugherty, 2014).

All four of these theories are useful for understanding family firms, but negotiating the often conflicting perspectives of members of the three family business systems is what makes family business leadership so different from leadership in other types of organizations. Leaders in public and non-family privately held businesses do not typically have to deal with input from family members when making business decisions. While leaders of non-family firms must meet owner expectations, those expectations are likely to be focused more exclusively on objective measures of financial returns. On the other hand, family business owners often have important non-financial socio-emotional goals (Gómez-Mejía et al., 2007) that may include producing goods or services for which they have a personal passion, reputation in the community, or providing employment for family members. Socio-emotional goals can conflict with business goals, but the family business leader who ignores them does so at his/her peril.

**Figure 1:**
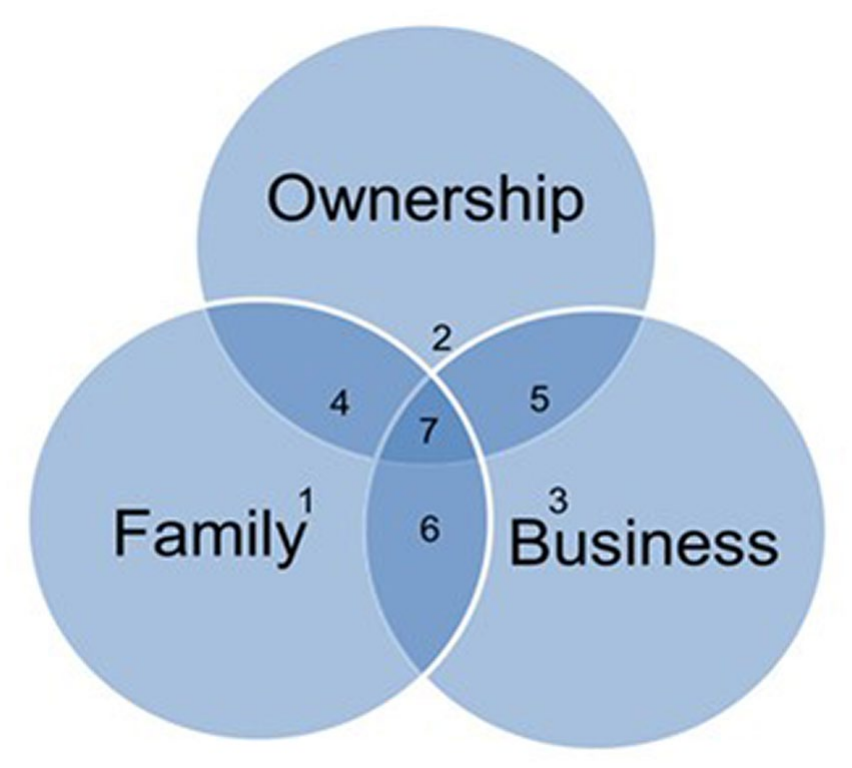
The three-circle model of family business (Gersick et al., 1997).

This study’s theoretical framework is influenced primarily by family business systems theory, as it suggests that family dynamics are likely to have an impact on how next-generation family members learn leadership behaviors. While the development of leadership skills for leaders in any context is influenced by the dynamics of the families in which they grew up, leaders of non-family businesses generally work in organizations where there are no family members present, affording them the opportunity to be exposed to different leadership paradigms and ways of doing business that may teach new leadership lessons. Furthermore, family relationships influence the leadership experiences of next-generation leaders of family firms throughout their careers, making it reasonable to think that what happens in the family system shapes the development of their leadership talent (Figure 1).

### Leadership theories and effective leadership

There is no single comprehensive theory that fully captures the complexity of leadership (Graen and Uhl-Bien, 1995). While Wren (2006) identifies 53 approaches to leadership research, complexity leadership theory and emotional and social intelligence, two of the leading theories, are highly relevant to leadership in a family business context and informed the study.

#### Complexity leadership theory

Family firms that have survived beyond the first generation are perfect examples of the kinds of complex organizations Marion and Uhl-Bien (2001) describe in their theory of leadership based on complexity theory. They assert that leaders of complex organizations are more effective if they create conditions conducive to the emergence of innovative solutions by fostering interaction among the subsystems within a larger complex system, as opposed to employing a more traditional command and control style of leadership. Complexity leadership theory suggests that next-generation leaders of family enterprises who foster positive interactions among the family, business, and ownership systems are likely to be more effective than those who only focus on one of those subsystems. This suggests next-generation leaders need to exercise a more collaborative style of leadership than the more autocratic style often employed by founders of family firms (Kets de Vries, 1993). That more autocratic leadership style may help entrepreneurs overcome the challenges of establishing a new business venture when it was more dependent upon his/her individual capabilities, but it is likely to become less effective as the business grows and becomes more complex. That suggests that next-generation leaders of family enterprises will need to develop a broader set of leadership skills than were required when the business was founded.

The complexity leadership theory provides support for the advice frequently offered by family business advisors who recommend that family firm leaders spend time and effort communicating openly and frequently with other family members working in and/or having ownership in the business to create a shared vision for the family enterprise and develop values, goals, and policies to guide decision making. Family business research confirms that developing a shared vision for the business among family owners is critical to family firm survival across multiple generations of ownership (Ward, 1988; Gersick et al., 1997; Ward, 2004, 2011; Poza and Daugherty, 2014; Zellweger, 2017) and is correlated with next-generation leader effectiveness and engagement with work (Miller, 2014). The more collaborative style of leadership needed to foster the development of that shared vision requires a leader with emotional and social intelligence, which is discussed next.

#### Competency-based emotional and social intelligence

How well leaders identify, understand, and control their own emotions, and read the emotions of those with whom they interact, determines the effectiveness of their leadership behavior and the quality of the relationships they create (Goleman et al., 2002). While technical skills are important to job performance at all levels, emotional and social intelligence is responsible for up to 90% of a leader’s effectiveness (Cherniss and Adler, 2000).

Competency-based emotional and social intelligence theory identifies 12 specific competencies that reflect the expression of emotional and social intelligence (see Table 1) and demonstrate self-awareness, self-management, social awareness, and relationship management skills (Goleman et al., 2002). While it is often assumed that “soft skills” like emotional and social intelligence competencies are innate or fixed in early childhood, they can be learned through impactful experiential learning (Cherniss and Adler, 2000; Goleman et al., 2002; Boyatzis and McKee, 2005).

**Table 1:** Emotional and social intelligence leadership competencies.

Self-awareness	Self-management	Social awareness	Relationship management
Emotional self-awareness	Emotional self-control	Empathy	Coach and mentor
	Adaptability	Organizational awareness	Inspirational leadership
	Achievement orientation		Influence
	Positive outlook		Conflict management
			Teamwork

Source: Boyatzis (2018).

Competency-based emotional and social intelligence theory, sometimes referred to as “mixed-model” or “behavioral” emotional intelligence, is one of three streams of emotional and social intelligence research, the others being “ability emotional intelligence” and “emotional self-efficacy.” Competency-based emotional and social intelligence predicts leadership effectiveness more reliably than the other streams of emotional intelligence research (O’Boyle et al., 2011) because it measures observable behaviors reflective of emotional and social intelligence. It has also been shown to have incremental validity above and beyond measures of personality characteristics and cognitive ability (O’Boyle et al., 2011). Because competency-based emotional and social intelligence predicts leadership effectiveness and work engagement in many different contexts (Boyatzis, 2018), and because effective leaders of family firms must successfully manage complex family relationships, it is at the core of the theoretical framework developed for this study.

#### Effective leadership

Leaders with true leadership talent inspire others to commit to a common goal (Hogan and Kaiser, 2005), like a shared vision for a family business. The most effective leaders create competitive advantage and produce desired organizational results by harmonizing intellectual, human, financial, and social capital (Boyatzis and McKee, 2005), skills necessary to achieve multi-generational success in a family enterprise (Ward, 1997). Because leadership effectiveness in a family firm requires achieving positive business results as well as fostering healthy relationships among family members who are involved in the operation and/or ownership of the firm, it is one of two dependent variables in the study.

### Work engagement

Studies of positive psychology identify work engagement as a primary indicator of well-being at work (Seppälä et al., 2009). It can be described as “a positive, fulfilling, work-related state of mind that is characterized by vigor, dedication, and absorption” (Schaufeli et al., 2002), and is the polar opposite of burnout (Schaufeli and Bakker, 2003). Next-generation leaders who are commited to the long-term survival of the family firms they lead demonstrate high levels of engagement by going above and beyond what is defined in their job descriptions (Dawson et al., 2015). Because they are self-aware, leaders high in emotional intelligence are less likely to experience burnout (Schaufeli and Bakker, 2003) and are more likely to engage in work that is well aligned with their values, thus generating an enormous amount of energy (Cherniss and Adler, 2000), an indicator of positive engagement with work. And because values are often instilled by one’s family, next-generation family members may find that work in the family firm is highly engaging and deeply meaningful.

Family business research has shown that family success positively influences business success, but that the opposite is not always true (Masuo et al., 2001). Work engagement is an indication that next-generation family members are benefitting from their work in the family firm at the same time their leadership is benefitting the family business. Work engagement is included as the second dependent variable of primary focus because it has such important benefits to family businesses and to the next-generation family members who lead them.

### Observer and self-ratings of emotional and social intelligence

The premise in this paper is that emotional and social intelligence competencies are supremely important in a family business context, as leaders must be attuned to both business *and* family relationships, which can be emotionally supercharged. Consequently, it is expected that several of the relationship management domains of emotional and social intelligence as identified by Boyatzis (2018) are likely to predict next-generation leader effectiveness and work engagement in a family firm. Those competencies include the ability to inspire others by creating a positive emotional tone and bringing out the best in people; fostering teamwork by being supportive, soliciting input, and encouraging cooperation; and mentoring and coaching by personally investing time and effort in developing others. Relationship-building skills like these are central to a family firm leader’s effectiveness in creating commitment to a shared vision for the family business, managing conflict and fostering cooperation among family members involved in the management and/or ownership of the family firm, and developing family members who will follow them in leadership roles, all of which are characteristics of family enterprises that survive through multiple generations of family ownership.

Studies have demonstrated that there is often a meaningful difference between self and other-ratings of emotional and social intelligence competencies. Self-ratings alone do not provide valid and reliable measures of emotional and social intelligence for research purposes (Boyatzis et al., 2017), observer ratings are more reliable indicators of leadership effectiveness (Boyatzis et al., 2015), and individuals are not very good judges of how others perceive their leadership behaviors (Tsui and Ohlott, 1988). Next-generation leaders of family firms who are also members of the business-owning family often do not receive accurate feedback on their leadership behaviors (Poza and Daugherty, 2014), suggesting that it is even less likely they will have accurate perceptions of their behaviors. Consequently, the study results are expected to show that observer ratings of next-generation leader emotional and social intelligence competencies will predict their leadership effectiveness but self-assessments will not, and the following hypotheses are advanced:

*H1: Observer ratings of next-generation family firm leader behaviors reflective of emotional and social intelligence competencies predict next-generation leader leadership effectiveness.*

*H2: Next-generation family firm leader self-ratings of leadership behaviors reflective of emotional and social intelligence competencies do not predict their leadership effectiveness.*

On the other hand, studies have shown that that self-perception of leadership behaviors is reflective of self-efficacy and that self-efficacy is predictive of engagement with work (Mauno et al., 2007; Xanthopoulou et al., 2007). A study of IT managers working in a variety of companies (Pittenger, 2012) demonstrated that self-rated emotional and social intelligence competencies predicted engagement with work. Consequently, it is hypothesized that:

*H3: Next-generation family firm leader self-ratings of leadership behaviors reflective of emotional and social intelligence competencies predict the degree to which they are engaged with their work in the family firm.*

### Responsibility and accountability

The literature on leadership and organizational behavior consistently identifies the degree to which leaders accept personal responsibility and are held accountable by others for their actions and decisions as hallmarks of leadership effectiveness. Taking personal responsibility includes accepting ownership of the results of one’s actions and decisions, being willing to face the truth even when it does not fit personal preferences, and avoiding making excuses for mistakes (Wood and Winston, 2005). Responsible leaders see themselves as stewards of the organizations they lead and keep stakeholders well informed, thus creating greater commitment to the goals of the organization (Fairholm, 2001). They accept responsibility for results, even if circumstances outside their control cause those results to be less than desirable (Conners et al., 1994; Manwaring, 1997; Kouzes and Posner, 2011; Kraines, 2011). Not only do they accept responsibility for what has happened in the past, but they also take responsibility for establishing a shared vision for the future as well (Kouzes and Posner, 2006; Kraines, 2011), one of the most important factors associated with family business longevity and success (Ward, 1988, 1997, 2004).

Accountability is “the leader’s willing acceptance of the responsibilities inherent in the leadership position to serve the well-being of the organization,” and includes the leader’s expectation that he or she will also be held accountable by others (Wood and Winston, 2005). In a family business context, this includes holding positions in the family firm with real responsibility and accountability, being held accountable by others, and experiencing the positive and negative consequences of one’s actions and decisions. An earlier qualitative study found that less effective next-generation family leaders often hold poorly defined roles in the family business and are not held accountable for the results of their leadership behaviors. That study also suggested that less effective next-generation family leaders are often shielded from the consequences of poor decision making and thus denied the opportunity of learning from mistakes (Miller, 2012).

It is expected that the degree to which the next-generation leaders in the study accept responsibility and are held accountable for their actions and decisions will influence others’ perceptions of their leadership effectiveness and their own engagement with work, leading to the following hypotheses:

*H4: The degree to which next-generation family firm leaders accept responsibility for their decisions and actions in the family business predicts their leadership effectiveness.*

*H5: The degree to which next-generation family firm leaders accept responsibility for their decisions and actions in the family business predicts the degree to which they are engaged with their work.*

*H6: The degree to which next-generation family firm leaders are held accountable for their decisions and actions in the family business predicts their leadership effectiveness.*

*H7: The degree to which next-generation family firm leaders are held accountable for their decisions and actions in the family business predicts the degree to which they are engaged with their work.*

### Family climate, open communication, and intergenerational authority

The influence of the family is what makes family firms different from businesses with other ownership structures. The primary focus of this study is to explore how the climate of the business-owning family affects the development of the leadership talent of next-generation family members who choose to work in the family firm and their engagement with that work. Family climate refers to the nature of family relationships and strongly influences the culture and performance of the family business (Björnberg and Nicholson, 2007). Open communication and intergenerational authority are two key dimensions of family climate as defined by Björnberg and Nicholson (2007).

Family business research identifies open and transparent communication in the family as one of the most important attributes of well-functioning family business systems (Gersick et al., 1997; Ward, 2004; Carlock and Ward, 2010; Poza and Daugherty, 2014). The literature documents its benefits to family business longevity, family relationships (Ward, 2004), effective family business governance (Pendergast et al., 2011), succession (Handler, 1994), conflict management, and a host of other factors. In business-owning families, open communication among family members includes taking time to listen to each other, openly expressing opinions, being honest and frank with each other, and openly addressing issues – good or bad (Björnberg and Nicholson, 2007).

Intergenerational authority refers to the degree of control exercised by the senior generation in a family. A family in which the older generation tends to set the rules and whose authority is not questioned by the younger generation is high in intergenerational authority. A family in which the younger generation participates in decision making and is encouraged to freely challenge the opinions of the senior generation is lower in intergenerational authority (Björnberg and Nicholson, 2007). A senior family leader who tries to exert too much control creates conflict that makes it more difficult for younger members of the family to differentiate themselves (Kerr, 1988) and develop their own leadership skills. This is important in family businesses because entrepreneurs who found or grow a family firm often employ this kind of autocratic leadership style (Kets de Vries, 1977; Kets de Vries, 1985).

Open communication and intergenerational authority in the family predict the degree to which there is a shared vision for the family firm (Miller, 2014), one of the primary predictors of multi-generational family firm survival (Ward, 1997). Open communication in the family has a positive effect and intergenerational authority has a negative effect on the existence of a shared vision for the family business. This study theorizes that open communication and intergenerational authority in the business-owning family will also affect the degree to which next-generation family leaders develop leadership skills and positively engage with their work in the family firm.

### Hypotheses related to family climate

We know that the emotional and social intelligence competencies that are so highly correlated with leadership effectiveness can be learned (Cherniss and Adler, 2000). We also know that learning leadership skills requires feedback from others (Mccall et al., 1988), and that it is difficult for next-generation family members working in the family business to get that feedback (Poza and Daugherty, 2014). A family that communicates openly is more likely to create an environment in which next-generation family members receive honest, useful feedback on their leadership behaviors than a family that avoids topics that can be difficult or sensitive to discuss. On the other hand, intergenerational authority is negatively correlated with open communication in the family (Björnberg and Nicholson, 2007), suggesting that senior family members who lead autocratically create an environment in which communication is shut down, thus limiting the opportunities for next-generation family leaders to receive feedback that helps them learn leadership skills. Consequently, the following hypotheses are advanced:

*H8: Open communication in the family positively influences the development of emotional and social intelligence competencies of next-generation leaders in family firms, which in turn positively affects their leadership effectiveness.*

*H9: Intergenerational authority negatively influences the development of emotional and social intelligence competencies of next-generation leaders in family firms, which in turn negatively affects their leadership effectiveness.*

Family communication theory identifies two common patterns of communication in family systems: conversation orientation and conformity orientation. Conversation orientation is defined as “the degree to which families create a climate in which all family members are encouraged to participate in unrestrained interaction” (Koerner and Fitzpatrick, 2002). Conformity orientation is defined as “the degree to which family communication stresses a climate of homogeneity of attitudes, values, and beliefs” (Koerner and Fitzpatrick, 2002). A family with a conversation orientation exhibits open communication as defined in this study. A family with a conformity orientation is likely characterized by a senior generation that is high in intergenerational authority. Adolescents from families with a conversation orientation have higher levels of self-esteem (Kelly et al., 2002). Those from families with a conformity orientation exhibit lower levels of self-esteem (Rangarajan and Kelly, 2006). We also know that self-perception of leadership behaviors is reflective of self-efficacy (Mauno et al., 2007; Xanthopoulou et al., 2007). Based on these theories, the following hypotheses are advanced:

*H10: Open communication in the family positively influences next-generation family firm leader self-ratings of leadership behaviors reflective of emotional and social intelligence.*

*H11: Intergenerational authority in the family negatively influences next-generation family firm leader self-ratings of leadership behaviors reflective of emotional and social intelligence.*

Next-generation leaders in family firms often have poorly defined roles with unclear responsibilities and authority (Kets de Vries, 1993), which in turn makes holding them accountable for results difficult. An earlier qualitative study showed that some senior leaders in family firms structure jobs for next-generation family members in a way designed to shield them from the consequences of their actions and decisions (Miller, 2012), thus signaling to others in the organization that they are not to be held accountable for the results they produce. It can also be difficult for senior family leaders who exhibit high levels of intergenerational authority, particularly entrepreneurs who have a high need to control (Kets de Vries, 1985), to delegate meaningful responsibilities to next-generation family leaders. In any of these situations, poorly defined jobs with unclear levels of authority make it challenging for next-generation leaders to assume responsibility for outcomes, and for others to hold them accountable.

It is logical to think that open communication in the business-owning family facilitates the creation of clearly defined roles with appropriate levels of authority for next-generation family leaders working in the business, which in turn encourages their acceptance of responsibility for their decisions and actions in those roles and makes it possible for them to be held accountable by others. Because intergenerational authority is negatively correlated with open communication in the family (Björnberg and Nicholson, 2007), it is also logical to think that next-generation leaders from families that exhibit higher levels of intergenerational authority are more likely to find themselves in poorly defined roles that make accepting responsibility and being held accountable less likely. The following hypotheses are proposed based on this logic and the cited research:

*H12: Open communication in the family positively influences the degree to which next-generation family firm leaders accept responsibility for their decisions and actions in the family business.*

*H13: Intergenerational authority negatively influences the degree to which next-generation family firm leaders accept responsibility for their decisions and actions in the family business.*

*H14: Open communication in the family positively influences the degree to which next-generation family* firm leaders are held accountable for their decisions and actions in the family business.

*H15: Intergenerational authority negatively influences the degree to which next-generation family firm leaders are held accountable for their decisions and actions in the family business.*

## Methods^1^

### Research design

A multi-rater cross-sectional research design was employed to measure the effects of family climate on the development of leadership skills among next-generation leaders in family businesses and the degree to wish they are engaged with their work. Next-generation leaders were defined as family members who held management positions in the family enterprise who were members of any generation other than the founding generation. A questionnaire was designed to capture the perceptions of a cross section of family and non-family members working in each family business that participated in the study. Each next-generation family leader and three to seven people familiar with his/her leadership behaviors (the “multi-raters”) filled out the survey.

Next-generation leaders answered questions about their own leadership behaviors, the climate of the business-owning family, and the nature of their engagement with their work in the family firm. The multi-raters answered the same set of questions about the next-generation leader’s leadership behaviors as well as a set of questions about the degree to which the next-generation leader accepts responsibility and is held accountable for his/her actions and decisions. The multi-raters also evaluated the next-generation leader’s leadership effectiveness. Multi-raters who were members of the business-owning family working in the family firm responded to the same set of questions about family climate that the next-generation leaders answered.

This multi-rater approach produces trait-context dyads that serve as latent variables in an aggregate multidimensional factor model (Batista-Foguet et al., 2019) and increases the accuracy of results. It is also the best *ex ante* procedure to avoid potential common method bias (Podsakoff et al., 2003), which can be introduced by including dependent and predictor variables in the same survey, and is considered the gold standard for evaluating leader behaviors as self-ratings alone are often unreliable and inflated (Taylor, 2014).

### Measurement development

Previously validated scales were used to measure the key concepts in the study and are described below. A set of five questions based on the results from an earlier qualitative study were included to measure accountability. Participants in the survey responded to questions using five-point Likert-type scales. A list of survey items is included in the Appendix.

#### Emotional and social intelligence

Observed leaderships behaviors reflect a leader’s emotional and social intelligence, which includes self-awareness, self-management, social awareness, and relationship management competencies (Boyatzis and McKee, 2005). Three scales from the Emotional and Social Competency Inventory – University Edition (Boyatzis and Goleman, 2007) were used to measure the relationship management competencies of the next-generation leaders who participated in the study, as the effect of family relationships on next-generation leader effectiveness and engagement with work is the purpose of the research (sample items in parentheses): *Coach and Mentor*, the extent to which the leader coaches and mentors others by investing time and effort in their development (“Personally invests time and effort in developing others.”); *Inspirational Leadership*, the extent to which the leader inspires and brings out the best in others, articulates a compelling vision, and creates a positive emotional tone (“Leads others by creating a positive emotional tone.”); and *Teamwork*, the extent to which the leader encourages participation of all team members, encourages cooperation, solicits input, and is supportive and respectful of team members (“Works well in teams by being supportive.”).

#### Family climate

Open communication and intergenerational authority in the business-owning family were measured using two scales, specifically designed for a family business context, from the Family Climate Scales (Björnberg and Nicholson, 2007). *Open Communication* measures how transparently a family communicates and includes listening, showing interest in each other’s opinions, and dealing forthrightly with issues of concern. *Intergenerational Authority* measures the degree to which the senior generation exercises power in the family, sets the rules of family conduct, and allows younger generations to question their authority.

#### Leadership effectiveness

Perceptions of next-generation leader effectiveness were measured using five items from the Leadership Effectiveness Scale (Denison et al., 1995). The Leadership Effectiveness Scale measures a leader’s overall leadership effectiveness and success, comparison to peers, performance as a role model, and the extent to which he/she meets leadership performance standards.

#### Responsibility

Next-generation leader acceptance of personal responsibility for his/her leadership behaviors and decisions and the results they produce was measured using 10 items from The Responsibility Scale (Wood and Winston, 2007).

#### Accountability

Five questions suggested by an earlier qualitative study that showed next-generation family members are sometimes shielded from the consequences of their actions and decisions and not held accountable to the same extent as non-family leaders (Miller, 2012) were added to the survey to determine if they formed a construct predictive of leadership effectiveness and/or engagement with work.

#### Work engagement

Next-generation leader engagement with their work in the family firm was measured using the nine-item version of the Utrecht Work Engagement Scale (Seppälä et al., 2009). The Utrecht Work Engagement Scale measures *Vigor*, the energy, effort, and persistence leaders invest in their work; *Dedication*, the sense of significance, enthusiasm, inspiration, pride, and challenge leaders derive from their work; and *Absorption*, how fully leaders concentrate on and become deeply engrossed in their work.

### Pre-testing, data collection, sample, and data screening

Survey questions were pre-tested using a Q-sort following guidelines suggested by Thomas and Watson (2002). The Q-sort demonstrated the face validity of all variables and was followed by two pilot tests of the online questionnaire.

An email list representing a wide variety of family-owned businesses was created from names provided by family business consultants, university-based family business centers, business trade organizations, and family business service providers. Approximately 9,537 email invitations were sent to leaders in those the family firms. 866 participants filled out the questionnaire for a response rate of 9.1%. Unfinished and incomplete surveys were removed from the database resulting in 567 usable surveys. Because multiple multi-raters were required for each next-generation leader included in the analysis, the database was further reduced to a matched set of 100 next-generation family leaders and 350 multi-raters for an average of 3.5 multi-raters per next-generation leader.

Next-generation leaders represented in the study were fairly evenly distributed across age ranges; 41% were G2 family members, 32% were G3, 17% were G4, 8% were G5 or higher, and 2% did not provide that information; all had at least a high school degree, 58% had a four-year college degree, and 32% had a master’s degree or higher; 51% were CEOs, 34% held other senior-level management positions, 10% held middle-level management positions, and 5% held entry-level management positions; 81% were male and 29% were female.

Family businesses represented in the study were also evenly distributed by size as measured by annual revenue. 29% had annual revenues under $25 million, 24% had annual revenues between $25 and $100 million, 26% had annual revenues between $101 and $250 million, and 21% had annual revenues of $250 million or more. 99% of the family firms in the study were privately owned. Respondent characteristics are shown in Table 2 and family business characteristics are shown in Table 3.

**Table 2:** Respondent characteristics.

	Matched Sample			
	Next-Generation Leaders		Multi-Raters	
	Number	Percent	Number	Percent
Sample size (*n*)	100		350	
Gender				
Male	81	81%	259	74%
Female	19	19%	88	25%
Missing	0	0%	3	1%
Age				
18–25	1	1%	11	3%
26–35	28	28%	55	16%
36–45	23	23%	84	24%
46–55	31	31%	97	28%
56–65	17	17%	84	24%
66+	0	0%	16	5%
Missing	0	0%	3	1%
Generation				
G1	0	0%		
G2	41	41%		
G3	32	32%		
G4	17	17%		
G5+	8	8%		
Missing	2	2%		
Education				
Less than high school	0	0%	0	0%
High school/GED	2	2%	27	8%
Some college	6	6%	53	15%
2-year college degree	2	2%	28	8%
4-year college degree	58	58%	154	44%
Master’s degree	27	27%	77	22%
Doctoral degree (PhD, EdD)	2	2%	0	0%
Professional degree (JD, MD)	3	3%	11	3%
Missing	0	0%	0	0%
Position in family business				
CEO	51	51%	17	5%
Other senior-level management	34	34%	190	54%
Middle-level management	10	10%	86	25%
Entry-level management	5	5%	16	5%
Non-management position	0	0%	39	11%
Missing	0	0%	2	1%
Family membership				
Family member			61	17%
Non-family member			288	82%
Missing			1	0%
Relationship with next-generation leader				
Immediate supervisor			22	6%
Other senior leader			36	10%
Direct report			144	41%
Other follower			45	13%
Peer			44	13%
Other relationship			51	15%
Missing			8	2%

**Table 3:** Family business characteristics.

Family business characteristics	Matched sample	
Sample size (*n*)	100	
Revenue		
Under $25 million	29	29%
$25–$50 million	9	9%
$51–$100 million	15	15%
$101–$250 million	26	26%
$251–$500 million	9	9%
$500 million+	11	11%
Missing	1	1%
Ownership		
Privately owned	99	99%
Public, but family controlled	0	0%
Public	0	0%
Other form of ownership	1	1%
Missing	0	0%

Data screening indicated that several variables exhibited negative skewness and/or kurtosis. Because multivariate analysis assumes normality of data, skewed variables were transformed by squaring or cubing which cured both skewness and kurtosis issues. All variables in the model exhibited homoscedasticity and linearity.

### Measurement model analysis

An exploratory factor analysis (EFA) was conducted with IBM SPSS 28.0.1.1 using principal components analysis with Promax rotation. Retained indicators loaded on their respective factors as expected, with values exceeding the .55 threshold recommended by Hair et al. (2010) as necessary for practical significance and indicator reliability for a sample size of 100. Cronbach’s alphas for all scales were above 0.70 indicating internal consistency and reliability (see Table 4). There was some cross-loading among several items from the emotional and social intelligence scales, which was expected as the scales were created from inductive studies of behavior anchored to performance criteria which resulted in a circumplex model of competencies. Consequently, it is assumed that some items as well as scales will have a high shared variance with others (Boyatzis et al., 2015).

**Table 4:** Measurement model results.

Constructs/Items	Mean	Std. dev.	Std. regression weights^1^	Cronbach’s alpha	Composite reliability	Average variance extracted	Maximum shared variance
Criteria^2^			>0.50	>0.70	>0.70	>0.50	<AVE
Accountability	65.42	17.31		0.79	0.83	0.63	0.59
ra_11_cub	103.50	26.67	0.83				
ra_12_cub	89.02	28.55	0.89				
ra_13	3.73	0.74	0.64				
Intergenerational authority	2.59	0.77		0.82	0.84	0.63	0.15
iaut_3	2.64	0.84	0.69				
iaut_4	2.84	0.97	0.75				
iaut_7	2.30	0.88	0.92				
leadership effectiveness	14.42	3.58		0.96	0.95	0.80	0.74
lev_1_sq	17.07	4.61	0.89				
lev_2	4.05	0.62	0.84				
lev_3_sq	17.27	5.08	0.92				
lev_4_sq	16.93	4.45	0.89				
lev_5_sq	16.81	4.63	0.93				
Multi-rater emotional intelligence	10.24	2.36		0.78	0.90	0.76	0.74
cm_cr	3.73	0.67	0.82				
il_cr	12.29	3.47	0.93				
tw_cr	14.70	3.40	0.86				
Next-gen self-rated emotional intelligence	3.91	0.56		0.82	0.82	0.60	0.49
ncm_cr	3.68	0.74	0.77				
nil_cr	3.92	0.61	0.85				
ntw_cr	4.13	0.61	0.70				
Open communication	7.57	2.38		0.87	0.87	0.57	0.24
oc_1	3.56	1.05	0.67				
oc_3	3.33	1.00	0.61				
oc_6_sq	14.11	5.10	0.84				
oc_7_sq	13.38	5.65	0.83				
oc_8	3.50	0.78	0.81				
Responsibility	46.83	12.61		0.93	0.93	0.73	0.59
ra_2_cub	92.50	26.82	0.88				
ra_5_sq	18.12	5.11	0.83				
ra_7_sq	18.38	4.36	0.85				
ra_9_cub	90.10	27.10	0.90				
ra_10_sq	15.07	5.45	0.83				
Work engagement	25.18	7.16		0.89	0.89	0.57	0.49
uwe_1	3.73	0.76	0.75				
uwe_2	3.96	0.78	0.75				
uwe_3_sq	19.27	5.92	0.84				
uwe_4	4.25	0.81	0.76				
uwe_5_sq	19.26	6.22	0.69				
uwe_7_cub	100.63	33.93	0.73				

Model fit				
Statistic	Threshold	Results		Reference
Chi square		594.20		
Degrees of freedom		458		
CMIN/DF	< 3.0	1.30		Carmines and McIver (1981)
CFI	0.92	0.95		Hair et al. (2010)
RMSEA	< 0.08	0.06		Hair et al. (2010)
PCLOSE	> 0.05	0.26		Hair et al. (2010)

1 *p* < 0.001 for all standardized regression weights;

2 Hair et al. (2010).

The EFA was followed by a confirmatory factor analysis (CFA). IBM AMOS 28.0.0 was used to create the measurement model and test for composite measure reliability and validity and model fit. Summated scales were created for each of the six emotional and social intelligence factors, as the items from each scale were unidimensional (loading strongly on a single factor) and represented the same concept as demonstrated in prior research (Boyatzis and Goleman, 2007; Boyatzis and Gaskin, 2010). These factors were labeled NCM (next-generation self-evaluated coach and mentor), NIL (next-generation self-evaluated inspirational leadership), NTW (next-generation self-evaluated teamwork), MRCM (multi-rater evaluated coach and mentor), MRIL (multi-rater evaluated inspirational leadership), and MRTW (multi-rater evaluated teamwork). Summated scales represent multiple aspects of a concept in a single measure to facilitate interpretation and reduce measurement error. To further facilitate model interpretation, and because coach and mentor, inspirational leadership, and teamwork are relationship management subscales of the broader emotional and social intelligence behavioral construct, second-order factors were created in the measurement model to represent the three next-generation self-rated emotional intelligence scales in a single factor (labeled NEI) and the three multi-rater emotional and social intelligence scales in another single factor (labeled MREI).

Composite reliability for the latent constructs in the model ranged from 0.82 to 0.95 (see Table 4), well above the recommended threshold of 0.70. Higher levels of reliability indicate lower levels of measurement error. Average variance extracted (AVE) ranged from 0.57 to 0.80, above the recommended threshold of .50, indicating that all constructs exhibit convergent validity. Average variance extracted for each construct was greater than its maximum shared variance (MSV) with any other construct demonstrating discriminant validity (Fornell and Larcker, 1981). Discriminant validity was further demonstrated by comparing the square root of AVE for each construct with its highest correlation with any other construct as shown in the correlations matrix in Table 5.

**Table 5:** Correlations matrix.

	NEI	LEV	OC	IAut	RESP	UWE	ACCT	MREI
NEI	0.77							
LEV	0.35	0.89						
OC	0.23	0.33	0.76					
IAut	−0.39	−0.31	−0.29	0.79				
RESP	0.40	0.76	0.49	−0.39	0.86			
UWE	0.70	0.31	0.33	−0.35	0.37	0.75		
ACCT	0.41	0.55	0.45	−0.38	0.77	0.51	0.79	
MREI	0.50	0.86	0.33	−0.30	0.75	0.30	0.51	0.87

Square root of AVEs on the diagonals. ACCT = accountability, IAut = intergenerational authority, LEV = leadership effectiveness, MERI = multi-rater emotional intelligence, NEI = next-gen self-rated emotional intelligence, OC = open communication, RESP = responsibility, UWE = work engagement.

The CFA demonstrated good model fit. CMIN/DF was 1.30, less than the maximum threshold of 3.0 recommended by Carmines and McIver (1981). CFI was.95, RMSEA was 0.06, and PCLOSE was 0.26, all of which exceeded the standards recommended by Hair et al. (2010) for models with sample sizes of less than 250 and more than 30 variables. See Table 4 for complete measurement model results.

Finally, a test for common method bias was conducted by adding a common latent factor to the model and comparing standardized regression weights of factor loadings with and without the common latent factor (Podsakoff et al., 2003). Differences in factor loadings in the models with and without the common latent factor were all significantly less than 0.20, indicating the lack of meaningful common method bias.

## Results

Covariance-based structural equation modeling (CB-SEM) in IBM AMOS 28.0.0 was used to assess the relationships among the constructs in the model and test the hypotheses. This method was well suited for the study because it enables the estimation of causal networks including direct and indirect effects simultaneously, a feature that proved important in demonstrating the indirect effects of family climate on next-generation leadership effectiveness and engagement with work. Before testing for the significance of path coefficients in the model, the predicator variables were tested for collinearity using IBM SPSS Statistics 28.0.1.1. Tolerance values below 0.20 and VIF values above 5 indicate potential collinearity problems. All predictor variables demonstrated tolerance and VIF values well within acceptable limits (see Table 6).

**Table 6:** Collinearity assessment.

Variable	Tolerance	VIF
Accountability	0.52	1.91
Intergenerational authority	0.80	1.25
Multi-rater emotional intelligence	0.49	2.03
Next-gen self-rated emotional intelligence	0.75	1.34
Open communication	0.78	1.29
Responsibility	0.34	2.91

### Statistical conclusions validity

The sample size of 100 is relatively small for the complex model used in the analysis of the data, so a post-hoc statistical power test was conducted. At both 0.05 and 0.01 significance levels, the model demonstrated more than 99% power levels for all endogenous constructs, indicating sufficient power to detect significant effects and to confidently reject relationships with insignificant effects.

### Significance of path coefficients

Standardized coefficients and *p*-values were calculated for statistically significant direct, indirect, and total effects to test the hypothesized relationships. Results are summarized in Tables 7–9 and discussed below.

**Table 7:** Significance testing results of structural equation model path coefficients direct effects.

Path	Standardized coefficient	*p* value	Effect size value (*f*^2^)	Effect size
ACCT → NEI	0.34	0.006	0.09	Small
ACCT → UWE	0.28	0.011	0.11	Small
IAut → NEI	−0.28	0.017	0.07	Small
IAut → RESP	−0.28	0.008	0.09	Small
MERI → LEV	0.66	<0.001	0.63	Large
NEI → UWE	0.58	<0.001	0.52	Large
OC → RESP	0.41	<0.001	0.20	Medium
RESP → ACCT	0.74	<0.001	1.48	Large
RESP → LEV	0.27	0.006	0.14	Small
RESP → MREI	0.75	<0.001	1.26	Large
Age → ACCT	0.28	<0.001	0.20	Medium

ACCT = accountability, IAut = intergenerational authority, LEV = leadership effectiveness, MERI = multi-rater emotional intelligence, NEI = next-gen self-rated emotional intelligence, OC = open communication, RESP = responsibility, UWE = work engagement.

**Table 8:** Significance testing results of structural equation model path coefficients indirect effects.

Path	Standardized coefficient	*p* value	Effect value (*v*^2^)	Effect size
ACCT → UWE	0.20	0.014	0.04	Small
IAut → ACCT	−0.21	0.013	0.04	Medium
IAut → LEV	−0.21	0.012	0.05	Medium
IAut → MREI	−0.21	0.015	0.04	Medium
IAut → NEI	−0.07	0.015	<0.01	No effect
IAut → UWE	−0.26	0.016	0.07	Medium
OC → ACCT	0.30	0.002	0.10	Large
OC → LEV	0.31	0.002	0.10	Large
OC → MREI	0.30	0.001	0.09	Large
OC → NEI	0.10	0.007	0.01	Small
OC → UWE	0.14	0.001	0.02	Small
RESP → LEV	0.49	0.003	0.24	Large
RESP → NEI	0.25	0.011	0.06	Medium
RESP → UWE	0.35	0.004	0.12	Large
Age → NEI	0.09	0.008	<0.01	No effect
Age → UWE	0.13	0.005	0.02	Small

ACCT = accountability, IAut = intergenerational authority, LEV = leadership effectiveness, MERI = multi-rater emotional intelligence, NEI = next-gen self-rated emotional intelligence, OC = open communication, RESP = responsibility, UWE = work engagement.

**Table 9:** Significance testing results of structural equation model path coefficients total effects.

Path	Standardized coefficient	*p* value
ACCT → NEI	0.34	0.013
ACCT → UWE	0.47	0.005
IAut → ACCT	−0.21	0.013
IAut → LEV	−0.21	0.012
IAut → MREI	−0.21	0.015
IAut → NEI	−0.35	0.007
IAut → RESP	−0.28	0.021
IAut → UWE	−0.26	0.016
MERI → LEV	0.66	0.003
NEI → UWE	0.58	0.005
OC → ACCT	0.30	0.002
OC → LEV	0.31	0.002
OC → MREI	0.30	0.001
OC → NEI	0.10	0.007
OC → RESP	0.41	0.002
OC → UWE	0.14	0.001
RESP → ACCT	0.74	0.003
RESP → LEV	0.76	0.002
RESP → MREI	0.75	0.003
RESP → NEI	0.25	0.011
RESP → UWE	0.35	0.004
Age → ACCT	0.28	0.004
Age → NEI	0.09	0.008
Age → UWE	0.13	0.005

ACCT = accountability, IAut = intergenerational authority, LEV = leadership effectiveness, MERI = multi-rater emotional intelligence, NEI = next-gen self-rated emotional intelligence, OC = open communication, RESP = responsibility, UWE = work engagement.

### Coefficients of determination

**Figure 2:**
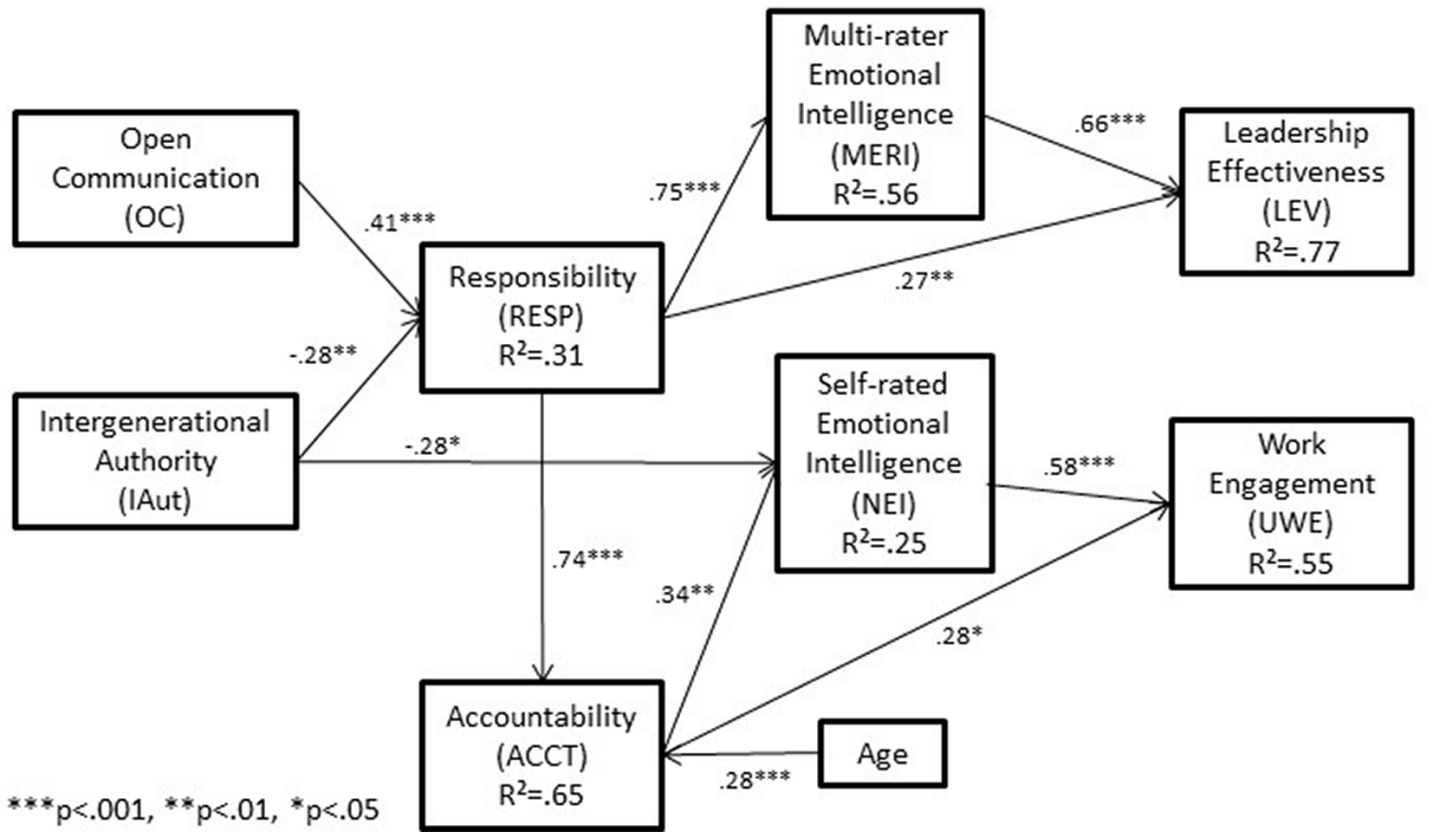
Final model.

Coefficient of determination (*R*^2^) indicates the amount of variance in a dependent variable explained by the predictor variables and is a commonly used measure of the explanatory power and predictive accuracy of a regression equation. Although there are no universally accepted standards for acceptable *R*^2^ values, Chin (1998) suggests that *R*^2^ values of 0.67, 0.33, and 0.19 indicate strong, moderate, and weak *R*^2^ values, respectively. *R*^2^ values for each of the six endogenous constructs are shown in the final model in Figure 2 (0.65 for Accountability, 0.77 for Leadership Effectiveness, 0.56 for Multi-rater Emotional Intelligence, 0.25 for Next-gen Self-rated Emotional Intelligence, 0.31 for Responsibility, and 0.55 for Work Engagement) and indicate that a meaningful amount of variance in the dependent variables of interest is explained.

### *f*^2^ effect size

The practical significance of the direct relationships in the structural equation model was determined by calculating *f*^2^ effect sizes using the formula:


$$f^{2}=\frac{R^{2}\;\mathrm{included}-R^{2}\mathrm{excluded}}{1-R^{2}\;\mathrm{included}}$$

*f*^2^ effect size measures the relative contribution of each exogenous variable in the model to the coefficient of determination (*R*^2^ value) of the endogenous variable it predicts. Cohen (1988) suggests that *f*^2^ effect size values of 0.02, 0.15, and 0.35 represent small, medium, and large effects, respectively. *f*^2^effect sizes for significant direct effects are shown in Table 7.

### *v*^2^ effect size

Effect sizes for indirect effects (*v*^2^) were calculated by squaring the standardized *v* effects (Lachowicz et al., 2018). Gaskin et al. (2023) suggest that *v*^2^ effect size values of 0.01, 0.04, and 0.09 represent small, medium, and large effects, respectively. *v*^2^ effect sizes for significant indirect effects are displayed in Table 8.

### Hypothesis testing results

Total effects (direct and indirect) of exogenous variables on endogenous variables are reported below and used to support or reject the study’s hypotheses as they provide the greatest insight (Hair et al., 2014). A summary of hypothesis testing results is provided in Table 10.

**Table 10:** Summary of hypothesis testing results.

Hypothesis	Standardized coefficient	Effect size	Support for hypothesis
*H1: Observer ratings of next-generation family firm leader behaviors reflective of emotional and social intelligence competencies predict next-generation leader leadership effectiveness.*	0.66***	*f*^2^ = 0.63	Yes
*H2: Next-generation family firm leader self-ratings of leadership behaviors reflective of emotional and social intelligence competencies do not predict their leadership effectiveness.*	−0.06 (ns)	n/a	Yes
*H3: Next-generation family firm leader self-ratings of leadership behaviors reflective of emotional and social intelligence competencies predict the degree to which they are engaged with their work in the family firm.*	0.58***	*f*^2^ = 0.52	Yes
*H4: The degree to which next-generation family firm leaders accept responsibility for their decisions and actions in the family business predicts their leadership effectiveness.*	0.27**	*f*^2^ = 0.14	Yes
*H5: The degree to which next-generation family firm leaders accept responsibility for their decisions and actions in the family business predicts the degree to which they are engaged with their work.*	0.35** (Indirect effect)	*v*^2^ = 0.12	Yes
*H6: The degree to which next-generation family firm leaders are held accountable for their decisions and actions in the family business predicts their leadership effectiveness.*	0.04 (ns)	n/a	No
*H7: The degree to which next-generation family firm leaders are held accountable for their decisions and actions in the family business predicts the degree to which they are engaged with their work.*	0.28*	*f*^2^ = 0.11	Yes
*H8: Open communication in the family positively influences the development of emotional and social intelligence competencies of next-generation leaders in family firms, which in turn positively affects their leadership effectiveness.*	0.31** (Indirect effect)	*v*^2^ = 0.10	Yes
*H9: Intergenerational authority negatively influences the development of emotional and social intelligence competencies of next-generation leaders in family firms, which in turn negatively affects their leadership effectiveness.*	−0.21* (Indirect effect)	*v*^2^ = 0.05	Yes
*H10: Open communication in the family positively influences next-generation family firm leader self-ratings of leadership behaviors reflective of emotional and social intelligence.*	0.10** (Indirect effect)	*v*^2^ = 0.01	Yes
*H11: Intergenerational authority in the family negatively influences next-generation family firm leader self-ratings of leadership behaviors reflective of emotional and social intelligence.*	−0.28*	*f*^2^ = 0.07	Yes
*H12: Open communication in the family positively influences the degree to which next-generation family firm leaders accept responsibility for their decisions and actions in the family business.*	0.41***	*f*^2^ = 0.20	Yes
*H13: Intergenerational authority negatively influences the degree to which next-generation family firm leaders accept responsibility for their decisions and actions in the family business.*	−0.28**	*f*^2^ = 0.09	Yes
*H14: Open communication in the family positively influences the degree to which next-generation family firm leaders are held accountable for their decisions and actions in the family business.*	0.30** (Indirect effect)	*v*^2^ = 0.10	Yes
*H15: Intergenerational authority negatively influences the degree to which next-generation family firm leaders are held accountable for their decisions and actions in the family business.*	−0.21* (Indirect effect)	*v*^2^ = 0.04	Yes

ns = non-significant.

*** *p* < 0.001.

** *p* < 0.01.

* *p* < 0.05.

*H1: Observer ratings of next-generation family firm leader behaviors reflective of emotional and social intelligence competencies predict next-generation leader leadership effectiveness.* As expected, the leadership effectiveness of next-generation leaders was strongly predicted by observer ratings (the multi-raters) of the degree to which their leadership behaviors reflect emotional and social intelligence (0.66, *p* < 0.001, *f*^2^ = 0.63), confirming the importance of next-generation leader emotional and social intelligence in a family business context.

*H2: Next-generation family firm leader self-ratings of leadership behaviors reflective of emotional and social intelligence competencies do not predict their leadership effectiveness.* H2 was supported as next-generation leader self-ratings of leadership behaviors had no significant relationship with observer evaluation of their leadership effectiveness (−0.05, *p* > 0.10) in the final model. However, when both observer-rated leadership behaviors and responsibility were removed from the model, there was a relationship between self-rated leadership behaviors and leadership effectiveness (0.35, *p* < 0.01), although that relationship only explained 12% (*R*^2^ = 0.12) of the variance in leadership effectiveness. This effect of self-rated behaviors on leadership effectiveness was fully mediated by observer-rated behaviors, indicating that self-rated emotional and social intelligence predicted leadership effectiveness only to the extent that it was consistent with observer-rated emotional and social intelligence. This finding suggests that leaders with accurate self-ratings are viewed as more effective than those whose self-ratings are not accurate.

*H3: Next-generation family firm leader self-ratings of leadership behaviors reflective of emotional and social intelligence competencies predict the degree to which they are engaged with their work in the family firm.* Next-generation self-ratings of their leadership behaviors reflective of emotional and social intelligence strongly predicted their engagement with work in the family firm (0.58, *p* < 0.001, *f*^2^ = 0.52) providing support for H3.

*H4: The degree to which next-generation family firm leaders accept responsibility for their decisions and actions in the family business predicts their leadership effectiveness.* H4 was also supported as the degree to which next-generation leaders accept responsibility for their actions and decisions strongly predicted their leadership effectiveness (0.79, *p* < 0.001), but this effect was partially mediated by the degree to which their leadership behaviors as observed by others reflected emotional and social intelligence, resulting in a smaller but still meaningful direct effect in the final model (0.27, *p* < 0.01, *f*^2^ = 0.14). This is one of the more important findings of the study and is interpreted in the discussion section of the paper.

*H5: The degree to which next-generation family firm leaders accept responsibility for their decisions and actions in the family business predicts the degree to which they are engaged with their work.* While there was a direct effect of the degree to which the next-generation leaders in the study accept responsibility on their work engagement (0.38, *p* < 0.001), this effect was fully mediated by the degree to which they are held accountable by others. A large indirect effect (0.35, *p* < 0.01, *v*^2^ = 0.12) through the accountability variable in the model remained, thus supporting H5. This finding has important implications for next-generation family firm leaders that is more fully explored in the discussion section of the paper.

*H6: The degree to which next-generation family firm leaders are held accountable for their decisions and actions in the family business predicts their leadership effectiveness.* H6 was not supported as the degree to which next-gen leaders are held accountable by others had no effect on their leadership effectiveness (0.04, *p* < 0.10).

*H7: The degree to which next-generation family firm leaders are held accountable for their decisions and actions in the family business predicts the degree to which they are engaged with their work.* H7 was supported as the degree to which next-gen leaders are held accountable had a small positive effect on their engagement with work (0.28, *p* < 0.05, *f*^2^ = 0.11). While this result may seem counterintuitive, it has particularly meaningful implications for next-gen leaders in family firms as further discussed in the discussion section of the paper.

*H8: Open communication in the family positively influences the development of emotional and social intelligence competencies of next-generation leaders in family firms, which in turn positively affects their leadership effectiveness.* While open communication in the family had a positive direct effect on observer-rated leadership behaviors reflective of next-generation leader emotional and social intelligence (0.34, *p* < 0.01), this effect was fully mediated by the degree to which next-generation leaders accept responsibility for their leadership actions and decisions. In the final model, open communication had strong positive indirect effects on observer-rated emotional and social intelligence (0.30, *p* < 0.001, *v*^2^ = 0.09) and on leadership effectiveness (0.31, *p* < 0.01, *v*^2^ = 0.10) through its direct effect on responsibility (0.41, *p* < 0.001, *f*^2^ = 0.20), thus providing support for H8. As open communication is one of the two family relationship variables hypothesized to have a meaningful effect on next-gen leader effectiveness and work engagement, this is one of the study’s key findings.

*H9: Intergenerational authority negatively influences the development of emotional and social intelligence competencies of next-generation leaders in family firms, which in turn negatively affects their leadership effectiveness.* Intergenerational authority had a negative direct effect on the observer-rated leadership behaviors reflective of emotional and social intelligence of next-generation leaders (−0.32, *p* < 0.01), but as with open communication, it was fully mediated by the degree to which next-generation leaders accept responsibility. In the final model, intergenerational authority had medium negative indirect effects on next-generation leader emotional and social intelligence (−0.21, *p* < 0.05, *v*^2^ = 0.04) and leadership effectiveness (−0.21, *p* < 0.05, *v*^2^ = 0.05), thus supporting H9. Intergenerational authority is the other family relationship variable hypothesized to have a significant effect on the leadership effectiveness of next-generation leaders, so this is also a key finding.

*H10: Open communication in the family positively influences next-generation family firm leader self-ratings of leadership behaviors reflective of emotional and social intelligence.* Open communication had a direct effect on next-generation self-evaluation of leadership behavior reflective of emotional and social intelligence (0.25, *p* < 0.05), but the effect was fully mediated by next-generation responsibility and accountability. The result was a small positive indirect effect (0.10, *p* < 0.01, *v*^2^ = 0.01) on next-generation self-evaluation of leadership behaviors, providing support for H10.

*H11: Intergenerational authority in the family negatively influences next-generation family firm leader self-ratings of leadership behaviors reflective of emotional and social intelligence.* Intergenerational authority had small negative direct (−0.28, *p* < 0.05, *f*^2^ = 0.07) and total effects (−0.35, *p* < 0.01) on next-generation self-evaluation of leadership behaviors, thus supporting H11. While the *f*^2^ effect size was small, this finding is considered meaningful within a family business context as explained in the discussion section.

*H12: Open communication in the family positively influences the degree to which next-generation family firm leaders accept responsibility for their decisions and actions in the family business.* Open communication in the family had a medium positive direct effect on the degree to which next-generation leaders accept responsibility for their actions and decisions (0.41, *p* < 0.001, *f*^2^ = 0.20) providing support for H12. This is one of the more important findings in the study, as it was through next-generation responsibility that open communication in the family affected all six of the endogenous variables in the model.

*H13: Intergenerational authority negatively influences the degree to which next-generation family firm leaders accept responsibility for their decisions and actions in the family business.* H13 is supported, as intergenerational authority had a small but meaningful negative direct effect (−0.28, *p* < 0.01, *f*^2^ = 0.09) on next-generation leader responsibility. As with open communication, this is a central finding of the study as intergenerational authority had direct or indirect effects on all six endogenous variables in the model.

*H14: Open communication in the family positively influences the degree to which next-generation family firm leaders are held accountable for their decisions and actions in the family business.* While open communication had a positive direct effect on the degree to which next-generation leaders are held accountable by others (0.47, *p* < 0.001), it was fully mediated by next-generation responsibility. The resulting strong indirect effect on accountability (0.30, *p* < 0.01, *v*^2^ = 0.10) in the final model provides support for H14.

*H15: Intergenerational authority negatively influences the degree to which next-generation family firm leaders are held accountable for their decisions and actions in the family business.* Intergenerational authority had a negative direct effect on the degree to which next-generation leaders are held accountable by others (−0.40, *p* < 0.01) that was fully mediated by next-generation leader responsibility. The resulting medium negative indirect effect (−0.21, *p* < 0.05, *v*^2^ = 0.04) supports H15.

### Controls

Age of next-generation leaders and size of the family business as measured by revenue were included as control variables. Size had no effect on any of the other variables in the model. Age was positively related to the degree to which next-generation leaders are held accountable for their actions and decisions (0.28, *p* < 0.001, *f*^2^ = 0.20) and had a small indirect effect on work engagement (0.13, *p* < 0.01, *v*^2^ = 0.02). This finding suggest that older next-generation leaders are held more accountable than younger leaders and are slightly more engaged with their work, which has meaningful implications for the development of next-generation family leaders in family firms and is interpreted more completely in the discussion section of the paper.

## Discussion

The primary purpose of the study was to examine how family climate influences factors that theory and prior research suggest affect the development of leadership skills and work engagement among next-generation family leaders in family businesses. Weak next-generation leadership is a major reason family firms fail to survive through multiple generations of family ownership (Ward, 1997), so a deeper understanding of what the family can do to help next-generation family members develop leadership skills can contribute to family firm longevity. Identifying factors that lead to positive engagement with work can help next-generation family members find meaning, purpose, and fulfillment in their work with the family enterprise.

The study resulted in three major findings: (1) the emotional and social intelligence of next-generation family business leaders drives their leadership effectiveness and partially mediates the relationship between the degree to which they take responsibility for their actions and decisions and their leadership effectiveness. (2) Family climate influences the degree to which next-generation family leaders accept personal responsibility for their actions and decisions and the results they produce, which predicts the degree to which their leadership behaviors reflect their emotional and social intelligence and leadership effectiveness. (3) Family climate influences the degree to which next-generation leaders are held accountable by others and evaluate their own leadership behaviors, which in turn affect how positively engaged they are with their work in the family firm. Interpretations of these results are provided next.

### Emotional and social intelligence drives leadership effectiveness

It was not surprising that observer-rated leadership behaviors reflective of emotional and social intelligence so strongly predicted next-generation leadership effectiveness (0.66, *p* < 0.001, *f*^2^ = 0.63), as emotional and social intelligence has been shown to predict leadership effectiveness in many other contexts (Goleman et al., 2002; Boyatzis, 2018). Three findings related to emotional and social intelligence, one of which was a surprise, are particularly relevant to family firms.

First, it was observer-rated rather than self-rated leadership behaviors reflective of emotional and social intelligence that predicted leadership effectiveness. This implies that there is a gap between the way the next-generation leaders in the study perceive their leadership behaviors and how others observe them, reflecting a lack of self-awareness. Self-awareness is the foundational emotional and social intelligence competency as it is necessary to learn or improve leadership skills (Goleman et al., 2002). This is important in a family business context because next-generation leaders who are also members of the business-owning family often do not receive accurate or frequent feedback on their leadership behaviors (Poza and Daugherty, 2014). Family members may be reluctant to communicate the need to improve leadership behaviors of next-generation family leaders for fear of damaging family relationships, or to “protect” developing next-generation family members from the consequences of poor leadership decisions (Miller, 2012). Non-family leaders in the family firm may also hesitate to counsel next-generation family members with the knowledge that the boss’s son, daughter, nephew, or niece may one day become their new boss. The conundrum is that next-generation leaders are unlikely to be motivated to improve if they are unaware of the gap between their self-perceptions and those of their observers. As discussed below, this study provides evidence that the nature of the family climate can positively or negatively influence the likelihood that they will receive the feedback they need to develop leadership competencies.

Second, as discussed earlier, three scales that measure relationship management competencies were used to represent emotional and social intelligence given the study’s focus on family relationships: *Coach and Mentor*, *Inspirational Leadership*, and *Teamwork*. Mentoring is important to the development of leadership skills in any context (Kram, 1988; Boyatzis, 2007; Ragins and Kram, 2007) and starts early in business-owning families as children are exposed to the family business and are often assigned age-appropriate tasks to perform in the family firm. In an earlier qualitative study (Miller, 2012), more effective next-generation family leaders often mentioned mentoring by another family member as important to their development as leaders. Inspirational leadership is critical to a family leader’s ability to develop and secure commitment to a shared vision for the family firm, one of the key determinants of family business survival through multiple generations of family ownership (Ward, 2004; Carlock and Ward, 2010; Poza and Daugherty, 2014). And teamwork is of supreme importance in a family firm because family members often work together in the business and committed non-family leaders are needed to complement the technical and leadership skills of family leaders. This study confirms that the degree to which next-generation leaders exhibit these relationship management competencies in their leadership behaviors is strongly related to how others evaluate their leadership effectiveness.

A surprise in the study was that the direct effect of next-generation leader acceptance of responsibility on their leadership effectiveness (0.79, *p* < 0.001) was strongly mediated by their observer-rated emotional and social intelligence, resulting in a smaller effect in the final model (0.27, *p* < 0.01, *f*^2^ = 0.14). This suggests that next-generation leaders who accept personal responsibility for their actions and decisions are viewed by others as effective leaders largely because they also exhibit leadership behaviors that reflect emotional and social intelligence competencies. Furthermore, the large effect of next-gen responsibility (0.75, *p* < 0.001, *f*^2^ = 1.26) on their observer-rated emotional and social intelligence suggests that one way next-generation family members learn leadership skills is by accepting personal responsibility, which is discussed next.

### Family climate influences the development of emotional and social intelligence competencies and leadership effectiveness

Context matters in learning the emotional and social intelligence competencies that influence leadership effectiveness (Cherniss and Adler, 2000; Boyatzis, 2006, 2008). Consistent with that theory, the results of the study demonstrate that family climate affects next-generation display of leadership behaviors reflective of emotional and social intelligence and their leadership effectiveness. In the final structural equation model, open communication in the family had large positive indirect effects on observer-rated next-gen emotional and social intelligence (0.30, *p* < 0.01, *v*^2^ = 0.09) and leadership effectiveness (0.31, *p* < 0.01, *v*^2^ = 0.10). Intergenerational authority had medium negative indirect effects on observer-rated next-gen emotional and social intelligence (−0.21, *p* < 0.05, *v*^2^ = 0.04) and leadership effectiveness (−0.21, *p* < 0.05, *v*^2^ = 0.05).

However, one of the most important findings in the study was that the mechanism through which family climate influences next-generation leader emotional and social intelligence and leadership effectiveness is through the degree to which the next gens accept responsibility for their actions and decisions. Next-generation leader responsibility fully mediated the direct effects of open communication (0.34, *p* < 0.01) and intergenerational authority (−0.32, *p* < 0.01) on observer-rated emotional and social intelligence. Open communication in the family had a medium positive direct effect on responsibility (0.41, *p* < 0.001, *f*^2^ = 0.20). Intergenerational authority had a small but meaningful negative direct effect on responsibility (−0.28, *p* < 0.01, *f*^2^ = 0.09). Responsibility had a very strong direct effect on observer-rated emotional and social intelligence (0.75, *p* < 0.01, *f*^2^ = 0.1.26). These findings suggest that next generation leaders learn emotional and social intelligence competencies by squarely facing leadership challenges and taking personal responsibility for the results of their leadership behaviors – and that the climate in the business-owning family influences the likelihood that they will assume that responsibility.

These results have important implications for business-owning families. We know that emotional and social intelligence competencies can be learned through practice (Cherniss and Adler, 2000; Boyatzis and McKee, 2005) and that positive support from trusted others facilitates the kind of changes necessary to learn leadership skills (Boyatzis, 2006, 2008). The study’s findings support the idea that business-owning families who create a positive family environment characterized by open communication make it easier for next-generation family members to take on the kind of personal responsibility that will help them learn emotional and social intelligence competences and effective leadership behaviors. The results also suggest that senior generation family leaders who find it difficult to delegate responsibility and share authority make it more difficult for next-generation family members to learn leadership skills by denying them opportunities to exercise age and experience-appropriate responsibilities.

There is an important message in the results for next-generation family leaders as well. Ultimately, they are responsible for their own development as leaders in the family firm. If they are fortunate enough to have a family that has established a pattern of open and transparent communication, then the opportunities for assuming responsibilities in the family business from which they can learn leadership skills may be greater, but they must still seek them out. On the other hand, if senior-generation family members exercise a more autocratic leadership style, one that is often observed in entrepreneurs who found family businesses (Kets de Vries, 1977; Kets de Vries, 1985), then next-gens will need to work harder to find opportunities for gaining leadership experience in or outside of the family firm. The effect of autocratic senior-generation family leaders on the degree to which next-generation leaders assume responsibility is negative and significant, but it is not so great that determined next gens cannot overcome it by taking responsibility for their own leadership development.

### Family climate influences next-generation leader accountability, self-evaluation, and work engagement

Accountability, the degree to which the next-generation leaders in the study are held accountable by others for their actions and decisions, and next-generation self-evaluation of their leadership behaviors turned out to be the major drivers of their engagement with work (*R*^2^ = 0.55). The degree to which next-generation leaders are held accountable by others had direct (0.28, *p* < 0.05, *f*^2^ = 0.11) and indirect (0.20, *p* < 0.05, *v*^2^ = 0.04) effects on how engaged they are with their work in the family firm.

These results are important because, as discussed earlier, next-generation family leaders often do not receive accurate feedback (Poza and Daugherty, 2014), and in some family businesses, they are actively shielded from the consequences of their leadership behaviors (Miller, 2012). Reticence to hold next-generation family members accountable may be founded on concerns that suffering the consequences of a leadership failure would damage a next-gen’s reputation, family relationships, or the reputation of the family business itself. Non-family leaders may be reluctant to hold them accountable for fear of retribution from other family members working in the business or from the next-generation leaders themselves. Or perhaps the reticence is based on the fear that holding a next-generation family leader accountable will cause them to be less engaged with their work, when in fact it has the opposite effect. Whatever the cause, the study’s results show that next gens engage more strongly with their work in the family firm when they know how their performance is perceived by others.

That idea is further supported by the finding that being held accountable has a small but meaningful direct effect on how next-generation leaders rate their own leadership behaviors (0.34, *p* < 0.01, *f*^2^ = 0.09), which in turn strongly affects their engagement with work (0.58, *p* < 0.001, *f*^2^ = 0.52). Self-rating of leadership behaviors is reflective of a leader’s self-efficacy (Mauno et al., 2007; Xanthopoulou et al., 2007), so these findings suggest that being held accountable is important to a next-gen’s development of self-confidence and the psychological benefits received from working in the family business.

Family climate affected next-generation leader accountability and work engagement in much the same way it affected observer-rated emotional and social intelligence and leadership effectiveness. In the final model, open communication in the family had a large positive indirect effect on the degree to which next-generation leaders are held accountable (0.30, *p* < 0.01, *v*^2^ = 0.10) and a small indirect effect on their engagement with work (0.14, *p* < 0.01, *v*^2^ = 0.02). Intergenerational authority had medium negative indirect effects on next-generation accountability (−0.21, *p* < 0.05, *v*^2^ = 0.04) and work engagement (−0.26, *p* < 0.05, *v*^2^ = 0.07). However, as with emotional and social intelligence, the degree to which next-generation leaders accept responsibility served as a mediator between the family climate variables and accountability. The direct effects of open communication (0.47, *p* < 0.001) and intergenerational authority (−0.40, *p* < 0.01) on next-generation leader accountability were fully mediated by responsibility. The degree to which next-generation leaders accept responsibility had a large direct effect on the degree to which they are held accountable by others (0.74, *p* < 0.001, *f*^2^ = 1.48), and a large indirect effect on work engagement (0.35, *p* < 0.01, *v*^2^ = 0.12). Next-generation acceptance of responsibility also had a direct effect on their engagement with work (0.37, *p* < 0.001), but it was fully mediated by the degree to which next-gens are held accountable by others.

These results suggest that next-generation leaders are more likely to be held accountable for their performance in the family firm, which in turns helps them develop self-confidence and more fully engage with their work, if they also take personal responsibility for their actions and decisions. And as discussed earlier, next gens are more likely to take personal responsibility if there is open communication in the business-owning family and less likely if members of the senior generation exercise a more authoritarian style of leadership.

The influence of family climate on next-generation work engagement is further demonstrated by the negative direct effect of intergenerational authority on next-gen self-evaluation of leadership behaviors (−0.28, *p* < 0.05, *f*^2^ = 0.07), resulting in a negative indirect effect on work engagement (−0.26, *p* < 0.05, *v*^2^ = 0.07). This suggests that more authoritarian senior generation family leaders inhibit the development of self-efficacy among next-gen family leaders making it more difficult for them to fully engage with their work in the family business.

The takeaway from this analysis of factors that affect next-generation work engagement is the same as for their development of leadership skills – what happens in the family matters. Business-owning families that foster open communication enhance a next-generation family member’s opportunity for positive engagement with their work in the family firm. Families that take time to listen and openly discuss important issues can help next-generation family members determine if working the family firm is the best career option for them and identify meaningful roles in the business for which they are qualified that match their aptitudes and interests. Senior members of the family who employ an autocratic leadership style are more likely to dictate roles in the family firm for which next-generation family leaders are not well suited and resist delegating genuine authority to them, making it less likely that they will find fulfillment in their work in the family business. And just as with the development of leadership skills, next-generation family leaders can increase the likelihood that they will derive positive psychological benefits from their work in the family business by identifying roles in the family business in which they can make meaningful contributions, taking personal responsibility for their performance, and asking others to hold them accountable. The research suggests that a next-generation family member who is unable to negotiate a role with real responsibility in the family business might be better off seeking employment outside the family firm if they want to develop their leadership skills and derive positive energy and fulfillment from their work.

### Controls

Age had a medium direct effect on next-generation accountability (0.28, *p* < 0.001, *f*^2^ = 0.20), and a small indirect effect on work engagement (0.13, *p* < 0.01, *v*^2^ = 0.02). These findings indicate that older next-generation leaders are held more accountable by others, and as a result, are somewhat more engaged with their work than younger leaders. Being held more accountable may be partly a function of level of responsibility, as older leaders are likely to hold higher management positions than younger leaders. Or it could simply mean that more is expected of older, more experienced next-generation leaders. Whatever the reason, it suggests that one way to help next-gen leaders derive fulfillment from their work in the family firm is to hold them more accountable earlier in their careers.

### Limitations

Next-generation family leaders who participated in the study did not comprise a strictly random sample because they voluntarily responded to email invitations, which limits the ability to generalize results across all family businesses (Shadish et al., 2002). There is also the possibility that the responses of the multi-raters reflect social desirability because they were nominated by the next-generation leaders who were the focus of the study. This risk is common to most multi-rater leadership studies and was reduced by the fact that survey responses were kept strictly confidential by the researcher. Ideally concepts like leadership effectiveness and work engagement would be measured with a longitudinal study since they develop over time, while this study reports the responses of participants at a particular point in time. Nonetheless, the reliability of the findings is enhanced because there is meaningful variation in the ages of the leaders in the study.

### Avenues for further research

The results of this study suggest that next-generation family leader acceptance of responsibility for their leadership behaviors affects their acquisition of the emotional and social intelligence competencies that drive their leadership effectiveness. The literature suggests other ways next-generation leaders develop leadership skills including challenging job assignments (Mccall et al., 1988), work experience outside the family firm (Danco, 1982; Ward, 1997), mentoring relationships (Ward, 1997; Boyatzis, 2007; Ragins and Kram, 2007), and formal leadership training (Mccall et al., 1988; Conger and Benjamin, 1999; Boyatzis and McKee, 2005; Boyatzis, 2008). A follow-up study on the effectiveness of specific experiences in helping next-generation leaders develop leadership skills in a family business context would be theoretically meaningful and useful for family business practitioners.

There is on ongoing debate in the literature about the accuracy of self-rated leadership behaviors reflective of emotional and social intelligence in predicting leadership effectiveness (Boyatzis, 2018). As reported in the results section, there was a relationship between self-rated leadership behaviors and leadership effectiveness (0.35, *p* < 0.01), but it was fully mediated by observer-rated leadership behaviors. This suggests that self-rated emotional and social intelligence predicts leadership effectiveness only to the extent that it is consistent with observer-rated emotional and social intelligence, and that leaders with more accurate self-ratings are viewed as more effective than those whose self-ratings are not as accurate. A study to compare the leadership effectiveness of leaders who have a more accurate view of their leadership behaviors with those who have a less accurate perception would contribute to our understanding of self-awareness on leadership effectiveness.

## Conclusion

The major story in the study is that the climate in business-owning families influences the development of the leadership skills of next-generation family members working in the family firm and the degree to which they derive inspiration, energy, enthusiasm, and pride from that work. That influence is expressed through the extent to which the next-generation family leaders themselves assume responsibility for their leadership behaviors and the results they produce. A family climate characterized by family members who listen to each other, openly express their opinions, and address difficult issues forthrightly encourages next-generation family members to take on the kind of responsibility that will help them develop as leaders and experience fulfillment from their work in the family business. A family climate characterized by a senior generation that tries to exert too much control by making all the rules and resisting delegation of some authority discourages next-generation family members from assuming responsibility for their leadership behaviors, making it more difficult for them to learn effective leadership skills and fully engage with their work in the family firm.

### Contributions

Much of the focus in family business research has been on engineering a smooth succession process, with a particular focus on the business and ownership systems. This study adds to our understanding of family business dynamics by demonstrating how what happens in the *family system* influences the development of the skills needed by next-generation family members to effectively lead the family enterprise and positively engage with their work. It adds to leadership literature by revealing how the development of emotional and social intelligence competencies is influenced by the nature of family relationships in a family business context. It also provides evidence that observer-rated emotional and social competencies is a more accurate predictor of leadership effectiveness than self-ratings, a debate which continues to play out in the literature (Boyatzis, 2018).

### Implications for practice

Family business owners who want to help next-generation family members prepare for leadership roles in the family firm should devote as much effort to building communication skills in the family as they do in building the business. Not only will that help next-generation family members develop the leadership skills they need to effectively lead the business in the future, but it will also make it more likely they will find fulfillment from a career in the family enterprise.

The study also suggests that the hard-charging authoritarian leadership style that entrepreneurs often employ in overcoming the challenges of creating a successful business becomes counterproductive when the time comes for helping next-generation family members develop the leadership skills they will need to effectively lead the family firm in the future. The study could not have been clearer in demonstrating that senior generation family leaders who set all the rules and exercise unquestioned authority impede the development of next generation family leaders and make it less likely that they will fully engage with their work in the family firm. As difficult as it may be, senior family business leaders who tend to lead autocratically should give up some of their control by identifying age and experience appropriate roles in the family business in which next-generation family members can exercise decision-making authority and be held accountable for results to help them develop the leadership skills and self-confidence necessary to become effective leaders.

Finally, the study demonstrates how important it is for next-generation family members working in a family firm to take personal responsibility for their own development as leaders. They should take the initiative to work with senior leaders in the family business to identify clearly defined roles with real responsibility in which they can serve and for which they have the necessary skills and experience. The nature of the family climate may make identifying those opportunities easier if it is characterized by open communication or more difficult if it is characterized by a high level of intergenerational authority, but the effects of intergenerational authority are not so great that they cannot be overcome with effort and lots of communication. If that kind of role cannot be identified, then it may be wise for next-generation family members to seek opportunities with real responsibility and accountability outside the family business to help them develop their leaderships skills, perhaps with an eye towards returning in the future.

## Data availability statement

The raw data supporting the conclusions of this article will be made available by the authors, without undue reservation.

## Author contributions

The author confirms being the sole contributor of this work and approved it for publication.

## Funding

Open access publication fee provided by Kenan-Flagler Business School, University of North Carolina at Chapel Hill.

## Conflict of interest

The author declares that the research was conducted in the absence of any commercial or financial relationships that could be construed as a potential conflict of interest.

## Publisher’s note

All claims expressed in this article are solely those of the authors and do not necessarily represent those of their affiliated organizations, or those of the publisher, the editors and the reviewers. Any product that may be evaluated in this article, or claim that may be made by its manufacturer, is not guaranteed or endorsed by the publisher.

## Footnotes

^1^This paper is based on the same data used for my earlier article on shared vision in a family firm and uses several of the same variables, although the focus is entirely different. Consequently, the methods section includes some of the same information as in the earlier article (Miller, 2014).
