# Supplementary material for: Family climate influences next-generation family business leader effectiveness and work engagement
Source: Front Psychol. 2023 Jun 15;14:1110282. doi: 10.3389/fpsyg.2023.1110282 (PMC10307980; doi:10.3389/fpsyg.2023.1110282)
Supplement: Supplementary file 1 [file Table_1.docx]

**Appendix**

**Survey Items**

| **Code** | **Family Climate - Intergenerational Authority** |
| --- | --- |
| iaut_1 | The younger generations try to conform with what older generation would want. |
| iaut_2 | The wishes of the older generation are obeyed. |
| iaut_3 | The authority of the older generation is not questioned. |
| iaut_4 | Family members of the older generation set the rules. |
| iaut_5 | We make decisions with every person having an equal say, regardless of seniority. (RC) |
| iaut_6 | Older and younger members have equal amounts of power. (RC) |
| iaut_7 | The word of the older generation is law. |
| iaut_8 | The younger generation is encouraged to freely challenge the opinions of the older generation. (RC) |
|  | Source: Family Climate Scales (Björnberg and Nicholson, 2007) |
|  | **Family Climate - Open Communication** |
| oc_1 | People don’t openly express their opinions. (RC) |
| oc_2 | We keep our views pretty much to ourselves. (RC) |
| oc_3 | We are polite rather than honest in how we communicate with each other. (RC) |
| oc_4 | We regularly talk about things that concern us. |
| oc_5 | People are not interested in each other’s opinions. (RC) |
| oc_6 | We take time to listen to each other. |
| oc_7 | We bring issues out in the open, good or bad. |
| oc_8 | We are frank with each other. |
|  | Source: Family Climate Scales (Björnberg and Nicholson, 2007) |
|  | **Accountability** |
| ra_11 | Has held positions within the family firm with real responsibility and accountability. |
| ra_12 | Has been held accountable for his/her decisions and actions in the family business. |
| ra_13 | Is often shielded from the consequences of his/her decisions and actions. (RC) |
| ra_14 | Receives accurate feedback on his/her performance. |
| ra_15 | Is held to the same standards as non-family employees. |
|  | Source: (Miller, 2012) |
|  | **Responsibility** |
| ra_1 | Demonstrates a sense of obligation to constituents when making decisions. |
| ra_2 | Accepts responsibility for his/her actions within the organization. |
| ra_3 | Clearly defines for constituents where his/her responsibilities end and theirs begin. |
| ra_4 | Provides constituents with safe ways to address grievances against him/her. |
| ra_5 | Avoids making excuses for mistakes. |
| ra_6 | Avoids blaming others for mistakes. |
| ra_7 | Is willing to face the truth, even when it does not fit his/her personal preferences. |
| ra_8 | Accepts responsibility for the future direction and accomplishments of the group. |
| ra_9 | Accepts ownership for the results of his/her decisions and actions. |
| ra_10 | Looks to himself/herself first when the group’s results are disappointing. |
|  | Source: The Responsibility Scale (Wood and Winston, 2007) |
|  | **Leadership Effectiveness** |
| lev_1 | Meets leadership performance standards. |
| lev_2 | Comparison to leadership peers. |
| lev_3 | Performance as a role model. |
| lev_4 | Overall leadership success. |
| lev_5 | Overall effectiveness as a leader. |
|  | Source: Leadership Effectiveness Scale (Denison et al., 1995) |
|  | **Work Engagement** |
| uwe_1 | At my work, I feel that I am bursting with energy. |
| uwe_2 | At my job, I feel strong and vigorous. |
| uwe_3 | I am enthusiastic about my job. |
| uwe_4 | My job inspires me. |
| uwe_5 | When I get up in the morning, I feel like going to work. |
| uwe_6 | I feel happy when I am working intensely. |
| uwe_7 | I am proud of the work that I do. |
| uwe_8 | I am immersed in my work. |
| uwe_9 | I get carried away when I’m working. |
|  | Source: Utrecht Work Engagement Scale (Seppälä et al., 2009) |
|  | **Emotional and Social Intelligence** |
|  | 15 items that measure coach and mentor, inspirational leadership, and teamwork. Used with permission. See Measurement Development section of paper for sample items. |
|  | Source: Emotional and Social Competency Inventory – University Edition (Boyatzis and Goleman, 2007) |
| (RC) = Reverse Coding | |
